# Effects of a 4400 km ultra-cycling non-competitive race and related training on body composition and circulating progenitors differentiation

**DOI:** 10.1186/s12967-022-03591-5

**Published:** 2022-09-04

**Authors:** Maria Teresa Valenti, Michele Braggio, Arianna Minoia, Gianluigi Dorelli, Jessica Bertacco, Francesco Bertoldo, Mattia Cominacini, Tonia De Simone, Maria Grazia Romanelli, Lekhana Bhandary, Monica Mottes, Luca Dalle Carbonare

**Affiliations:** 1 Department of Medicine, Section of Internal Medicine, University of Verona, Piazzale Scuro, 10, Policlinico G.B. Rossi, 37134 Verona, Italy; 2 Department of Neurosciences, Biomedicine and Movement Sciences, University of Verona, 37100 Verona, Italy; 3 Flaskworks, LLC, 38 Wareham St, Boston, MA 02118 USA

**Keywords:** Ultra-cycling, Training, Progenitor cells, Transcription factors, Body composition, Fat mass, Aerobic capacity, Biochemical parameters

## Abstract

**Background:**

NorthCape4000 (NC4000) is the most participated ultra-endurance cycling race. Eight healthy male Caucasian amateur cyclists were evaluated: (a) before starting the preparation period; (b) in the week preceding NC4000 (after the training period); (c) after NC4000 race, with the aim to identify the effects of ultra-cycling on body composition, aerobic capacity and biochemical parameters as well as on the differentiation of progenitor cells.

**Methods:**

Bioelectrical impedance analysis (BIA) and dual energy x-ray absorptiometry (DEXA) assessed body composition; cardiopulmonary exercise test (CPET) evaluated aerobic capacity. Differentiation of circulating progenitor cells was evaluated by analyzing the modulation in the expression of relevant transcription factors. In addition, in vitro experiments were performed to investigate the effects of sera of NC4000 participants on adipogenesis and myogenesis. The effects of NC4000 sera on Sestrins and Sirtuin modulation and the promotion of brown adipogenesis in progenitor cells was investigated as well. Two-tailed Student’s paired-test was used to perform statistical analyses.

**Results:**

We observed fat mass decrease after training as well as after NC4000 performance; we also recorded that vitamin D and lipid profiles were affected by ultra-cycling. In addition, our findings demonstrated that post-NC4000 participant’s pooled sera exerted a positive effect in stimulating myogenesis and in inducing brown adipogenesis in progenitor cells.

**Conclusions:**

The training program and Ultra-cycling lead to beneficial effects on body composition and biochemical lipid parameters, as well as changes in differentiation of progenitor cells, with significant increases in brown adipogenesis and in MYOD levels.

## Background

In recent years, a trend towards greater participation and better performance times in ultra-endurance cycling has been recorded, despite the increasing average age of participants [1, 2].

Ultra-cycling performances lead to important energy deficit, especially in master athletes, due to the physiological impossibility of maintaining a positive balance with caloric replenishment [3]. In fact, a prolonged energy deficit is the main mechanism involved in fat mass (FM) loss [4].

Discrepancies regarding weight loss and possible lean mass alterations in ultra-endurance emerge in the literature. FM and fat-free mass (FFM) were analyzed before the Swiss-cycling Marathon with the aim to characterize competitive ultra-cyclers [5]. Variations in these parameters were evaluated in races like XX Alps 2004, with decrease in both FM and FFM using skin-folds method [6]. The same results were observed using Dual-Energy X-ray Absorptiometry (DEXA) after an 8,835 km mountain bike race [7]. However, others highlighted an increase in lean mass after an 883 km 6-days cycling stage race [8]. Bioelectrical Impedance analysis (BIA) showed a reduction in FM and an increase in body mass and FFM after 1000 km laboratory-based nonstop cycling, probably due to liquid retention [9]. Total body water also increased after a 600 km ultra-cycling race, which lead to a reduction in FM but no decrease in skeletal muscle mass (MM) measured with skin-folds method [9].

Little is known whether FM and lean mass modifications persist after a short period of recovery and if these modifications are related to aerobic capacity (e.g. VO2max): in fact, some authors reported a VO2max decrease [7] while others reported no significant changes [10].

Ultra-endurance might also lead to blood biochemical parameters alterations, including liver enzymes, creatinine, and lipid profile; anyhow in general, only case reports on ultra-cycling [7] and data on a small number of elite athletes [11] are available. On the other hand, few studies have reported that low vitamin D levels may be associated with musculoskeletal injuries, and that supplementation could improve performance [12], but to the best of our knowledge, nobody has evaluated possible vitamin D alterations in ultra-endurance cycling performance.

Despite the growing interest in amateur sports, few studies have been conducted in this field aiming at the evaluation of ultra-endurance exercise effects on health. In particular, changes in FM and LM and their possible association with markers of muscle and adipose cells metabolism following ultra-endurance races are not deeply understood. A comprehensive analysis on training parameters and aerobic capacity may help to clarify these modifications. Therefore, the aim of this study was to evaluate the effects of both preparation and competition of a 4400 km ultra-cycling adventure on body composition, aerobic capacity and biochemical parameters, and their possible association with circulating progenitors commitment in order to evaluate the impact of such physical exercise in the prevention of degenerative diseases.

Awareness of the impact of such physical efforts may set amateur sportsmen towards a more mindful and personalized training.

## Methods

### The race

NorthCape4000 (NC4000) is the most participated ultra-endurance unsupported cycling adventure.

The 4th edition began July, 24th, 2021. It covered 4,400 km and an elevation gain of 40,000 m and consisted of an unsupported, non-drifting race, where participants arrived to North-Kapp (NRW), starting from Rovereto (ITA), passing through 4 mandatory checkpoints (Lake Balaton, Krakow, Riga, Rovaniemi). Organizers set a completion time limit of 22 days.

Monitored temperature fluctuations were between 32° and 7 °C.

### Participants

Eight healthy male amateur Caucasian cyclists (47.5 ± 13.5 years) who attended the NC4000 4th edition were contacted via social media and underwent clinical evaluation, bioelectrical impedance analysis (BIA), cardiopulmonary exercise testing (CPET) and venipuncture for blood samples collection, before the preparation period (BPP), from 21st Dec 2020 to 2nd Mar 2021), the week before NC4000 (BN) and up to 10 days after NC4000 (AN). Dual energy x-ray absorptiometry (DEXA) was taken BN and AN. All data reported during NC4000 were assessed at the visit AN.

The study was approved by the ethical committee of Azienda Ospedaliera Universitaria Integrata of Verona, Italy (number 1538; Dec. 3, 2012; local ethical committee of Azienda Ospedaliera Integrata di Verona). The study design and methods comply with the Declaration of Helsinki.

Cyclists gave their voluntary written consent before the procedures.

### Clinical evaluation and questionnaires

All subjects underwent clinical evaluation to exclude any condition that might have altered performance or laboratory tests results.

BPP and BN training was reported as the mean of number of sessions per week, total kilometers per week and hours per week in the previous 3 months.

Sleeping hours per night, coffee and alcohol intake (measured as standard drinks) were also reported.

### Body composition measures

Total body dual-energy X-ray absorptiometry (DEXA) was taken BN and AN to measure total (FM) and segmental fat (truncal FM), visceral adipose tissue (VAT) and lean mass (LM) (QDR Discovery Acclaim; Hologic, Waltham, MA, USA). DEXA's body composition analysis were the same as previously described [13].

Tetra-polar dual frequency BIA (InBody 120; Cerritos, USA) was used BPP, BN and AN to measure weight and to estimate FM, FFM, and MM. Impedance measurements were performed after voiding, in the morning after at least 24 h of rest, with the athlete barefoot, standing in an upright position, in light cycling wear. Two measures were taken each time for each subject and the mean value was reported. Body height was measured to the nearest 0.5 cm while subjects stood barefoot.

### Blood samples collection

Blood samples were collected in the morning BPP, BN, AN. Biochemical parameters considered in this study were: ALT, AST, creatinine, 25-hydroxy vitamin D (Liaison^®^ Assay, DiaSorin, Italy), total cholesterol, HDL, LDL, triglycerides concentrations.

### Circulating progenitor cells (CPCs)

We isolated CPCs from heparinized blood, as previously reported [14]. After the collection of peripheral blood mononuclear cells (PBMCs) by a gradient centrifugation (800 × *g* for 30 min at 20 °C), we removed the unwanted hematopoietic cells by using a RosetteSep antibody cocktail (Stemcell Technologies Inc., Vancouver, Canada), according to manufacturer’s instructions. Collected cells were washed in phosphate-buffered saline (PBS) and phenotype analyses were performed as previously described [14].

### In Vitro* Myogenic Differentiation.*

Human Skeletal muscle cells (SKMC) were obtained from PromoCells (C-12580; PromoCell, GMBH Heidelberg, Germany). SKMCs were cultured with Skeletal Muscle Cell Growth medium (Low serum) (C-23060PromoCell, GMBH Heidelberg, Germany) and differentiated by using Skeletal Muscle Differentiation Medium Supplements (C-23061; PromoCell, GMBH Heidelberg, Germany), as we previously reported [15]. Differentiating SKMCs were cultured with or without pooled sera of participants at 5% final concentration. The medium was changed every 3 days after initial plating.

### RNAs extraction and reverse transcription

146b and 34a miRNAs were extracted from PBMCs by using miRNeasy kit (Qiagen, Hilden, Germany), accordingly to manufacturer’s instruction. Total RNA from CPCs or differentiating cells was extracted with the “RNeasy^®^ protect mini kit” (Qiagen, Hilden, Germany), following the manufacturer’s protocol and quantified by a Qubit™ 3 fluorometer using a “Qubit™ RNA HS assay kit (Invitrogen, Carlsbad, USA). Two micrograms of the extracted RNAs were reverse transcribed by using the TaqMan microRNA Reverse Transcription kit (Thermofisher Corporation, Waltham, MA, USA) or the First Strand cDNA Synthesis kit (GE Healthcare, Little Chalfont, UK), as previously reported [15]. RNA and cDNA samples were stored at − 80 °C.

### Real time RT–PCR

For miRNA or RNAs expression analysis we used Real-time PCR using TaqMan Universal PCR Master Mix (Thermofisher Corporation, Waltham, MA, USA) and TaqMan pre-designed probes for each gene (RUNX2, hs00231692_m1; sestrin1, hs00902782_m1; miR-146b-5, PN4440886; miR-34a, PN4427975; U6 snRNA, 001,973; MYOD, Hs00159528_m1; PPARG2, hs01115513_m1; ACTB, Hs99999903_m1). Ct values for each reaction were calculated by using TaqMan SDS analysis software (Applied Biosystems; Foster City, California, USA) and the relative gene expression levels between different samples, was analyzed by using the 2^ − ΔΔCT^ method, as previously reported [14].

### Western blotting

Ripa buffer (Thermo Fisher Scientific, Waltham, MA,USA) was used to extract the protein and concentrations were calculated with BCA assay (Thermo Scientific, Waltham, MA, USA) as previously reported [14]. Protein samples were diluted in Laemmli’s sample buffer (Biorad, CA, US) and heated for 5 min at 95 °C, and then separated by sodium dodecyl sulfate–polyacrylamide gel electrophoresis (SDS PAGE). Proteins were transferred onto polyvinylidene difluoride (PVDF) membranes (Thermo Fisher Scientific, Waltham, MA, USA). PVDF membranes were probed with the primary (antiMyoD (MA1-41,017; Thermo Scientific, Waltham, MA, USA); β ACTIN (BA3R; Thermo Scientific, Waltham, MA, USA); sirtuin (PA5-23,063; Invitrogen, Waltham, MA, USA); SESN1 (PA5-98,142; Invitrogen, Waltham, MA, USA); SESN2 (ab-178518; Abcam, Cambridge, MA); p53 (2524; Cell Signaling Technology, Danvers, Massachusetts, USA); p21 (M7202; Dako, Denmark A/S); UCP1 (ab-10983; Abcam, Cambridge, MA)) and secondary antibodies Anti-mouse (7076; Cell Signaling Technology) and Anti-rabbit (7074, Cell Signaling Technology). Signals were detected with a chemiluminescence reagent (ECL, Millipore, Burlington, MA, USA) and images were recorded using a LAS4000 Digital Image Scanning System (GE Healthcare, Little Chalfont, UK). Densitometric analysis was performed as we previously reported [16].

### Cardiopulmonary exercise test (CPET)

CPET was carried out on a cycle ergometer with clip-on pedals (Monark^®^, LC6 Novo; Vansbro, Sweden), using a gas analyzer (COSMED^®^ Quark PFT; Milan, Italy). Ventilation per minute (VE), oxygen uptake (VO2), carbon dioxide production (VCO2), first (VT1) and second (VT2) ventilatory thresholds and maximal oxygen uptake (VO2max) were assessed. VT1 was determined by V-slope method and VT2 by ventilatory equivalent method. Subjects performed a ramp test protocol with increments of 15, 20 or 25 W per minute until volitional exhaustion. The protocol was chosen based on age and training, as to reach at least 8 min of incremental phase [17] and a 6–20 modified Borg scale was used [18]. Cadence ranged between 70 and 100 rpm, as chosen by each subject. Each subject performed the same CPET protocol BPP, BN, AN. Heart rate (HR) and electrocardiogram (ECG) were constantly monitored. CPET was considered maximal when the following criteria were achieved: (a) a plateau > 20 s in the oxygen consumption (VO2) versus exercise intensity relationship (b) respiratory exchange ratio (RER > 1.10), (c) HR [≥ 85% of (220-age)], (d) a rating of perceived exhaustion (RPE) of 19–20 on the Borg modified scale. After the test, all respiratory data were averaged at 10-s intervals to determine VO2max, taken as the highest average value.

The above-mentioned measurements were made in the following order during the same day, in the morning: blood samples, body composition measures (BIA and DEXA), CPET.

Subjects avoided heavy physical effort at least 24 h before the tests, slept at least 6–8 h the night before and had a light meal at least 2 h before the tests. Sera were stored at − 80 °C until use.

### Statistical analysis

Results were expressed as mean ± SD. Statistical analysis was assessed by two-tailed Student’s paired-test. Differences were considered significant with p < 0.05. For in vitro data, analyses were applied to experiments carried out at least three times. We used SPSS for Windows, version 22.0 (SPSS Inc., Chicago, IL, USA) to analyze the data.

## Results

Anthropometric characteristics of cyclists are reported in Table 1. 6 out of 8 participants completed NC4000 within 22 days: finishing time was 21.24 ± 8.41 days, which consisted of 229.85 ± 68.76 km per day. Bike plus bags weight at start was 20.44 ± 4.30 kg. None of the cyclists had experienced a multi-day ultra-cycling race before. None of them followed a professional training or a specific diet, there were no significant differences in dietary habits of participants. None of them were vegetarian or had food allergies or intolerances.

**Table 1:** - Characteristics of the cyclists and training for NC4000

	BPP	BN	p
Age (years)	47.5 ± 13.5	–	
Height (cm)	178.8 ± 5.1	–	
Weight (kg)	79.25 ± 8.98	77.68 ± 8.16	0.042*
BMI (kg/m^2^)	24.79 ± 2.50	24.31 ± 2.34	0.040*
Training sessions	3.13 ± 0.84	3.50 ± 1.20	0.80
Time per week (hours)	11.25 ± 3.54	11.25 ± 2.44	0.999
Distance per week (km)	241.25 ± 148.75	259.38 ± 113.34	0.448
Sleeping hours	7.13 ± 1.00	6.81 ± 1.13	0.279

*BMI* body mass index; *BPP* before preparation period; *BN* before NC4000 performance (post training period); *AN* after NC4000 performance

*p < 0.05

Reported individual training program did not change significantly between BPP and BN.

### Effects of the training program before ultracycling performance

Weight and FM_BIA_ were significantly reduced after the training for NC4000 (Table 2). Coffee intake and sleeping hours were the same BPP and BN. A total of 6 cyclists used supplements between BPP and BN evaluation, and in particular saline solutions (5 out of 8), branched-chain amino acids (BCAAs, 3 out of 8), maltodextrins (2 out of 8), Vitamin B complex (1 out of 8), Vitamin C (1 out of 8) and Omega3 (1 out of 8).

**Table 2:** Pre- and post-training body composition variables (BIA)

	BPP	BN	p
FM_BIA_ (kg)	13.81 ± 6.41	11.18 ± 5.05	0.014*
FFM_BIA_ (kg)	65.44 ± 4.37	66.50 ± 4.99	0.055
FFM_BIA_ (%)	83.05 ± 6.07	85.94 ± 85.94	0.007*
MM_BIA_(kg)	37.06 ± 2.39	37.73 ± 2.80	0.060

*BIA* Bioelectrical impedance analysis; *BPP* before preparation period; *BN* before North-Cape4000; *FM* fat mass; *FFM* fat free mass; *MM* muscular mass

*p < 0.05

Total cholesterol and LDL decreased significantly between BPP and BN, while HDL and triglycerides, liver enzymes and creatinine did not change after training. Vitamin D levels rose significantly between BPP and BN (Table 3).

**Table 3:** - Pre- and post-training biochemical parameters

	BPP	BN	p
Vitamin D (ng/mL)	20.35 ± 7.19	32.38 ± 8.18	< 0.001*
Creatinine (mg/dL)	0.94 ± 0.11	0.91 ± 0.07	0.500
AST (IU/L)	25.13 ± 8.08	23.75 ± 4.30	0.500
ALT (IU/L)	23.38 ± 6.57	21.25 ± 5.75	0.190
Total cholesterol (mg/dL)	224.13 ± 34.48	204.88 ± 25.73	0.013*
LDL (mg/dL)	134.88 ± 28.62	117.75 ± 23.96	0.010*
HDL (mg/dL)	66.5 ± 14.60	63.63 ± 11.46	0.280
Triglycerides (mg/dL)	107.38 ± 48.25	117.50 ± 40.14	0.450

*ALT* alanine amino transferase; *AST* aspartate amino transferase; *HDL* high density lipoproteins; *LDL* low density lipoproteins

*p < 0.05

Absolute and relative VO2max, as well as ventilatory thresholds didn't increase after training for NC4000 (Table 4).

**Table 4:** VO2max and ventilatory thresholds pre- and post-training

	BPP	BN	p
VO2max (L/min)	3871.50 ± 649.36	3792.25 ± 365.42	0.492
VO2max (mL/min*kg^−1^)	49.61 ± 10.51	49.43 ± 7.64	0.884
VT1 (mL/min*kg^−1^)	35.85 ± 7.73	35.06 ± 6.23	0.738
VT2 (mL/min*kg^−1^)	45.94 ± 10.57	45.49 ± 6.43	0.803

*VO2max* maximal oxygen uptake; *VT1* first ventilatory threshold; *VT2* second ventilatory threshold

### Effects of NC4000 on body composition, ventilator parameters and biochemical markers

Subjects were evaluated AN 5.8 ± 2.2 days of recovery.

Reported water intake during NC4000 was 6.13 ± 2.37 L per day. Sleeping hours decreased significantly during NC4000 (6.81 ± 1.13 vs 5.25 ± 1.60; p = 0.027), while coffee intake did not change (2.75 ± 1.49 vs 3.50 ± 1.39; p = 0.307), as well as alcoholic intake (0.69 ± 0.59 vs 0.63 ± 0.79; p = 0.844).

All the subjects took supplements while ultra-cycling: the most used were saline solutions (7 of 8) and branched-chain amino acids (BCAAs, 6 of 8), followed by Vitamin B complex (5 of 8), Vitamin C (4 of 8), maltodextrins (2 of 8) and carnitine and Omega3 (1 of 8). Six cyclists used nonsteroidal anti-inflammatory drugs (NSAIDs) due to musculoskeletal injuries, none of them related to direct trauma. Illness or other significant health complaints were not reported.

Weight (77.68 ± 8.16 kg) and BMI (24.31 ± 2.34 kg/m^2^) did not decrease after NC4000, compared to BN. However, FM decreased, measured both with DEXA and BIA. DEXA also revealed a significant decrease in segmental fat, and an increase in relative LM. BIA highlighted an increase in FFM% but not in MM or absolute FFM (Table 5).

**Table 5:** - Body composition Before NC4000—After NC4000

	BN	AN	p
FM_DEXA_	18.24 ± 5.58	15.97 ± 4.54	0.002*
VAT (g)	474 ± 267	378 ± 184	0.019*
TF (kg)	8.63 ± 3.87	7.09 ± 3.06	0.003*
LM (kg)	55.65 ± 3.05	56.44 ± 3.17	0.122
LM (%)	72.94 ± 4.71	75.31 ± 4.18	< 0.001*
LAM (kg)	8.25 ± 0.34	8.34 ± 0.48	0.283
FM_BIA_ (kg)	11.18 ± 5.05	9.21 ± 3.75	0.019*
FFM_BIA_ (kg)	66.50 ± 4.99	66.95 ± 5.24	0.450
MM_BIA_ (kg)	37.73 ± 2.80	37.88 ± 2.97	0.676
FFM_BIA_ (%)	85.94 ± 5.06	88.05 ± 4.23	0.013*

*AN* after NC4000; *BIA* bioelectrical impedance analysis; *BN* before NC4000; *DEXA* dual energy x-ray absorptiometry; *FM* fat mass; *LAM* lean appendicular mass; *TF* truncal fat; *VAT* visceral adipose tissue

*p < 0.05

Serum 25-hydroxy Vitamin D decreased significantly after the race. Lipid profile showed an increase in triglycerides and a reduction in LDL concentration, whereas there was no variation in total cholesterol and HDL levels. Creatinine decreased significantly after NC4000. Liver enzymes concentrations did not change after ultra-cycling (Table 6).

**Table 6:** Pre- and post-race biochemical parameters

	BN	AN	p
Vitamin D (ng/mL)	32.38 ± 8.18	29.00 ± 5.81	0.042*
Creatinine (mg/dL)	0.91 ± 0.07	0.85 ± 0.10	0.021*
AST (IU/L)	23.75 ± 4.30	49.00 ± 46.23	0.162
ALT (IU/L)	21.25 ± 5.75	57.00 ± 65.48	0.153
Total cholesterol (mg/dL)	204.88 ± 25.73	201.63 ± 21.33	0.652
LDL (mg/dL)	117.75 ± 23.96	99.75 ± 17.12	0.047*
HDL (mg/dL)	63.63 ± 11.46	66.00 ± 9.89	0.462
Triglycerides (mg/dL)	117.50 ± 40.14	175.25 ± 64.16	0.004*

*ALT* alanine amino transferase; *AST* aspartate amino transferase; *HDL* high density lipoproteins; *LDL* low density lipoproteins

*p < *0.05*

After NC4000, there was a slight tendency in reduction of maximal oxygen uptake as well as oxygen uptake at first and second ventilatory threshold, but it was not statistically significant (Table 7).

**Table 7:** VO2max and ventilatory thresholds pre- and post-NC4000

	BN	AN	p
VO2max (L/min)	3792.25 ± 365.42	3690.25 ± 331.89	0.178
VO2max (mL/min*kg^−1^)	49.43 ± 7.64	48.74 ± 5.58	0.587
VT1 (mL/min*kg^−1^)	35.06 ± 6.23	34.20 ± 5.15	0.564
VT2 (mL/min*kg^−1^)	45.49 ± 6.43	45.23 ± 4.28	0.816

*VO2max* maximal oxygen uptake; *VT1* first ventilatory threshold; *VT2* second ventilatory threshold

### Effects of training and NC4000 on mesenchymal stem cells commitment

**Fig. 1:**
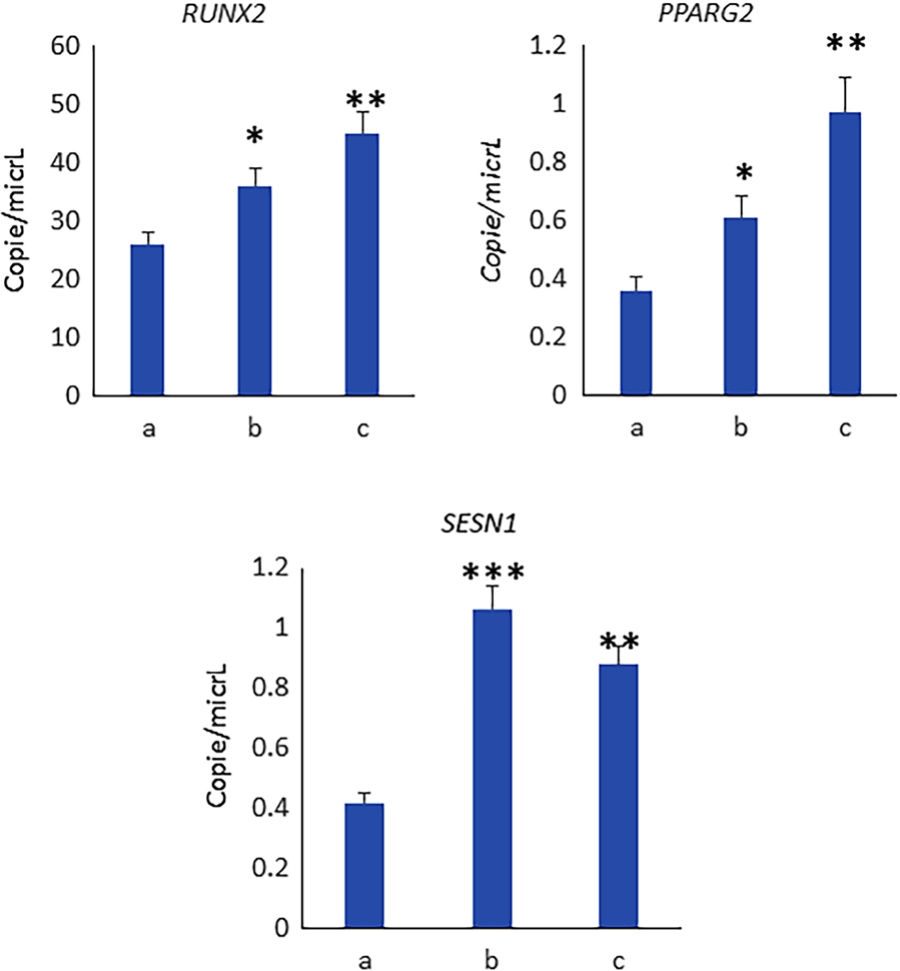
RUNX2 and PPARG2 were upregulated in BN (**b**) and AN (**c**) compared to BPP (**a**). BN (**b**) and AN (**c**) performance upregulated SESN1 expression in circulating MSCs: **a** (BPP, before the preparation period); **b** (BN, the week before NC4000); AN (c, 10 days after NC4000). *p < 0.05; **p < 0.005; ***p < 0.001

In order to identify the effects of training and NC4000 on Mesenchymal Stem Cells (MSCs) commitment, we analyzed circulating MSCs for the expression of the transcription factors RUNX2 and PPARG2, involved in osteogenesis and adipogenesis, respectively. As shown in Fig. 1, the osteogenic and adipogenic transcription factors, RUNX2 and PPARG2 were upregulated BN and AN. The training as well as ultra-cycling, upregulated Sestrin 1 (SESN1) expression in circulating MSCs.

### Training increases sestrins and sirtuin levels in SK muscle cells

**Fig. 2:**
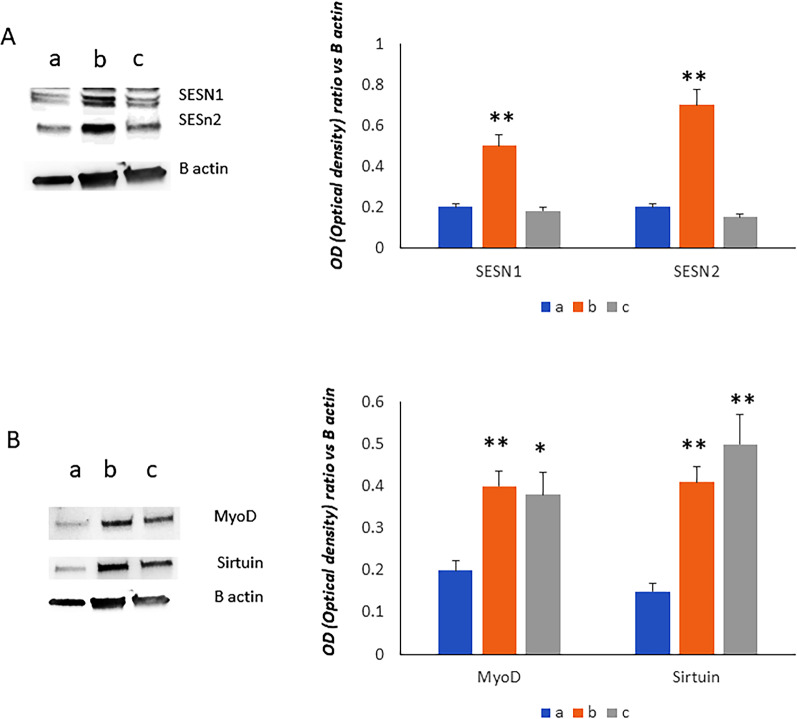
Sestrins increased after the training (b, BN) while NC4000 performance (c, AN) restored their protein levels. MyoD levels increased after the training (b, BN) and NC4000 performance (c, AN). SIRT1 levels increased in cells cultured in the presence of sera collected in subjects after the training (b, BN) and NC4000 performance (c, AN). **a** (BPP, before the preparation period); **b** (BN, the week before NC4000); AN (c, 10 days after NC4000). *p < 0.05; **p < 0.005

Since Sestrins regulate muscle stem cell homeostasis [19], we analyzed the SESN1 and SESN2 levels in SK muscle cells cultured in the presence of sera collected BPP, BN and AN. As shown in Fig. 2, both sestrins increased after the training while ultra-cycling restored their protein levels. Accordingly, MyoD levels were increased after the training and NC4000 (Fig. 2). Considering that sirtuin 1 (SIRT1), in the presence of MyoD, is involved in the regulation of cellular metabolism, we analyzed the protein levels of SIRT1 in SK muscle cells cultured in the presence of sera collected before, after the training and following ultra-cycling. As shown in Fig. 2B, SIRT1 levels increased in cells cultured in the presence of sera collected BN and AN.

### Training and ultra-cycling counteract adipogenesis aging and increases brown adipogenesis

**Fig. 3:**
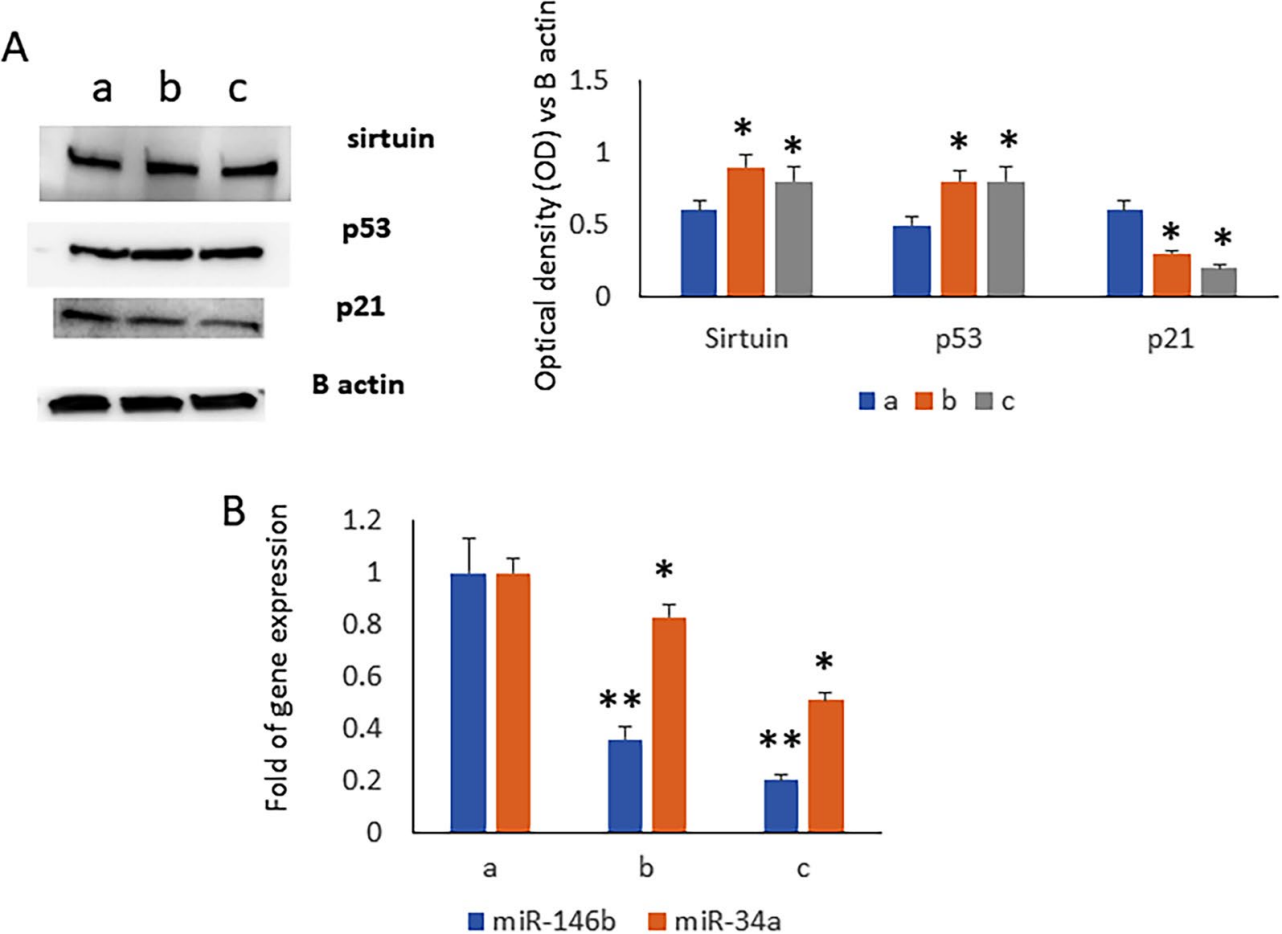
Increased levels of Sirtuins and p53 and reduced levels of p21 as well of miR-146b expression were observed in MSCs in presence of sera collected in subjects after the training (b, BN) and NC4000 performance (c, AN). **a** (BPP, before the preparation period); **b** (BN, the week before NC4000); AN (c, 10 days after NC4000). *p < 0.05; **p < 0.005

**Fig. 4:**
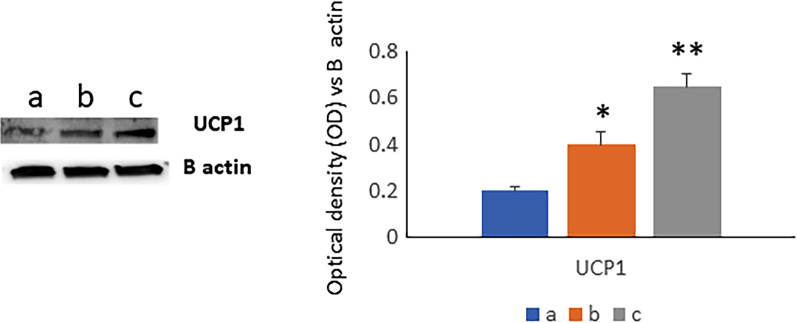
The levels of UCP1, associated to the brown adipose tissue (BAT) differentiation increased after the training as well as after ultra-cycling. **a** (BPP, before the preparation period); **b** (BN, the week before NC4000); AN (c, 10 days after NC4000). *p < 0.05; **p < 0.005

In order to evaluate the effects of training and ultra-cycling on adipogenesis, we cultured MSCs during adipogenesis in the presence of sera collected BPP, BN and AN. We observed increased levels of sirtuin in adipogenic differentiating cells after training and NC4000 (Fig. 3). In addition, we observed increased levels of p53 and reduced levels of p21, a target gene of p53 associated to senescence (Fig. 3). The observed reduction of p21 suggests a reduced transcriptional function of p53. Interestingly, it has been reported that p21 deficiency caused a suppression of adipocyte differentiation [20]. To explain the increased levels of sirtuin, we investigated the expression of its targeting microRNA, miR146b as shown in Fig. 3B, miR 146b was downregulated in MSCs in presence of sera collected in subjects after the training and NC4000 performance. As it has been reported that sirtuin plays an important role in the induction of brown adipose tissue (BAT) associated genes [21], we evaluated the levels of the uncoupling protein (UCP1) in differentiating cells. As shown in Fig. 4, UCP1 levels increased after the training as well as after ultra-cycling.

## Discussion

Ultra-Endurance cycling is a very challenging performance, typically defined as a race that is over 100 miles. This experience can take place on almost any surface and with any sort of bike, but its duration is the main characteristic. Ultra-cycling is associated with a multitude of physiological consequences. Although in recent years an increased interest in ultra-endurance sports has arisen, few studies have evaluated the impact of this physical effort in the modulation of biochemical parameters and in the commitment of progenitor cells. This is a relevant aspect as progenitor cell homeostasis is a pivotal factor in the pathogenesis of degenerative diseases and all factors influencing and maintaining this homeostasis are potential target in disease prevention. In addition, another original aspect is that the present study has been performed on amateur competitive cyclists subjected to lower training volumes than professional cyclists [22]. Therefore, in our study, we highlighted the possible connection between changes in body composition, adipogenesis and myogenesis.

Fat mass decrease could be explained by the well-known high-energy deficit caused by ultra-endurance events, especially in amateur cyclists [3, 23]. In this study, for the first time we evaluated the possible driver genes and transcriptional factors involved in this mechanism. In fact, physical training promotes the differentiation of Mesenchymal Stem Cells [24]. As previously reported [14], we observed increased expression of RUNX2 in cMSCs after the training, suggesting their osteogenic commitment. The increased RUNX2 expression in cMSCs was maintained AN. We also observed increased levels of PPARG2 after the training as well after NC4000. These findings could suggest an increase of adipogenic commitment too. However, the increased PPARG2 expression is in agreement with its role in regulating insulin sensitivity and the utilization of glucose to maintain energy homeostasis [25]. Accordingly, we also observed in cMSCs the expression of SESN1gene, coding for a small stress-inducible protein which has been demonstrated to be able to improve insulin sensitivity [26]. Interestingly, for the first time, we investigated the expression of Sestrin in circulating progenitor cells. The increased expression of SESN1 in progenitors highlights the effective role of physical exercise in degenerative diseases considering the protective role of sestrin [27]. Therefore, given the role of sestrins in the modulation of muscle metabolism following exercise, we investigated SESN1 and SESN2 protein levels in skeletal muscle cells treated with serum collected before and after training as well after NC4000. Interestingly, we observed increased levels of SESN1 and SESN2 in SK muscle cells treated with sera collected after the training while both SESN1 and SESN2 levels returned to baseline in cells treated with serum after NC4000 performance. Considering that sestrins have protective activity against diseases affecting the musculoskeletal system [27], it can be considered that training is useful to counteract sarcopenia, muscle atrophy, osteopenia and arthritic pathologies.

Despite some studies reported a decrease in free fat mass (FFM) and fat mass (FM) after ultra-cycling [6, 7], we observed a decrease in FM and maintenance of muscular and lean mass: this was consistent also with training to ultra-cycling. Previous different results may be explained by the hydration status of the subjects studied and the scheduled evaluation in the immediate post-competition time, which causes localized edema in the main muscle groups analyzed, an effect that can alter DEXA results [28].

Furthermore, we did not observe alterations in ALT and AST concentrations, which are implicated in muscular and subsequent liver damage following strenuous exercise. We can speculate that these parameters were higher immediately after NC4000 [29] and reparation of muscle fibers was ongoing. These findings were not altered by modifications in coffee and alcohol intake during NC4000. Super-compensation in storage of skeletal muscle glycogen and fluids could have affected the lean mass measurements AN, considering that cyclists were evaluated about one week after recovery from the NC4000 and that serum creatinine levels were significantly reduced. Additional evidence comes from Knechtle et al., who stated that ultra-cycling does not alter skeletal muscle mass [30].

Therefore, we investigated the role of training and ultra-cycling on skeletal muscle cells. It has been reported that the expression of SESN1 is induced during myotube differentiation [26]. Accordingly, we observed increased levels of sestrins as well of MYOD in SK muscle cells treated with sera collected after the training. The increased levels of MYOD have been maintained also after NC4000 performance, despite their tendency to decrease. Sirtuin1, is a histone/protein deacetylase which regulates the caloric restriction-mediated longevity [31]. It has been demonstrated that in the presence of MyoD, SIRT1 is able to induce positive self-regulation of the expression of the peroxisome proliferator activated receptor-γ co-activator-1α (PGC-1alpha), a master regulator of genes involved in the regulation of metabolism [32]. In skeletal muscle, exercise induces PGC-1α expression probably in response to decreased ATP levels [33]. Therefore, the increased levels of MYOD and Sirtuin 1 observed in SK muscle cells treated with sera collected after training suggest the role of physical exercise in driving muscle-specific gene expression and metabolism.

We also evaluated whether aerobic capacity, in terms of VO2max and ventilatory thresholds were affected by individual training and ultra-cycling. Interestingly, VO2max, VT1 and VT2 were not modified. Our explanation for these results is that our subjects already had good aerobic capacity and that their training habits did not change before NC4000. No changes were highlighted after ultra-cycling, despite other studies having reported a general increase in aerobic capacity [10]. With regard to this aspect, our subjects were better trained and during ultra-cycling pedaled an average of more than 220 km per day, which is much more than what has been previously reported. Even if aerobic capacity was not affected by training and NC4000, skeletal muscle metabolism was positively stimulated.

Sirtuin 1 levels also increased in MSCs during adipogenic differentiation in the presence of sera collected after the training and ultra-cycling. The increased levels of Sirtuin 1 reduced the transcriptional activity of p53. We observed reduced levels of p21, a downstream target gene of p53, even if p53 levels increase after training and NC4000. Moreover, it has been demonstrated that SIRT1 can prevent the transcriptional activity of p53 by deacetylation process [34]. Importantly, it has been demonstrated that SIRT1 activation during adipogenesis promotes the transcription of brown adipose tissue specific genes [35]. In addition, SIRT1 is also involved in the downregulation of WAT gene expression [36].

Thus, we observed increased levels of the uncoupling protein1 (UCP1), uniquely expressed in BAT cells, in MSCs during adipogenesis in the presence of sera collected after training. Importantly, the increase of UCP1 was more pronounced in the presence of sera collected AN. These results highlighted the relationship between BAT and the substantial reduction in truncal and visceral adipose tissue (VAT) as a marker of negative energy balance observed after NC4000.

VAT has important implications on whole cardiovascular risk and sequestration of vitamin D: thus, we evaluated lipid profile and Vitamin D levels. We observed a significant decrease of total cholesterol and LDL during the preparation period. LDL decreased also after NC4000, which may be related to a higher demand of fatty acids for beta-oxidation. On the other hand, HDL remained at high levels during the study, without substantial modifications also after NC4000. These results are consistent with the increase UCP1 expressed in activated BAT, that is confirmed as a lipid profile modulator [37]. This result is in contrast with ultramarathon running and underlines the differences in these sports [38]. Vitamin D levels and lipid profile were not altered by supplements, because only one cyclist reported the use of carnitine and another one omega 3.

We found a clear increase in triglycerides concentration after NC4000: others had explained this aspect by a catecholaminergic stimulus induced by exercise; this result seems to be in contrast with what we found about other lipid profile parameters, but this may be due to increased hepatic VLDL–triglyceride production or to increased circulating non-esterified fatty acids (NEFAs) after the competition [10] which mask the plasma triglyceride lowering.

Serum 25-hydroxy Vitamin D increased significantly between BPP and BN: this could be explained by a better sun exposition during spring and summer months [12].

However, Vitamin D concentrations slightly decreased after NC4000, and this outcome may be justified by the consumption of adipose deposits in a short period or by the delay in determining vitamin D levels after the end of the competition, confirming the complex relationship between ultra-endurance and vitamin D metabolism [39].

By considering that the activation of BAT, as well as the body composition modulation, had been suggested to reduce obesity related diseases, the aim to further investigate the role of strenuous physical exercise in relation to cardiovascular and degenerative diseases appears particularly interesting. In order to identify epigenetic factors involved in the increase of sirtuin1, we analyzed the expression of miR targeting sirtuin1. Accordingly, we observed reduced miR146b and miR34a in cells treated with sera collected after BN and AN. Interestingly, MiR-146b has been suggested as a regulator of human visceral adipogenesis and its expression is altered in human obesity (40), confirming the importance of the modulation of this metabolic pathway in preventing cardiovascular and degenerative diseases.

Therefore, our study, for the first time, evaluates the functional biochemical parameters with the modulation of changes in the progenitor cells and analyzes, as in the case of sestrins, the impact of physical exercise on molecules involved in the the pathogenesis of degenerative diseases. The results of our study, conducted on healthy subjects, can therefore be useful to better understand the impact of physical performance related to ultra-endurance activity in the commitment of progenitor cells and therefore in a possible prevention of degenerative diseases.

## Conclusions

The training program leads to beneficial effects on body composition and biochemical lipid parameters, as well as a switch in brown adipogenesis and substantial modification in cellular processes related to SESN1 and SESN2 expression, even when oxygen uptake and ventilatory thresholds are unaltered in trained amateur cyclists.

Ultra-cycling does not alter body weight or aerobic capacity but produces an acute and prolonged energy imbalance that induces a reduction in fat mass, changes in commitment of MSCs, with significant increase in brown adipogenesis and UCP1 and a maintenance of MYOD levels in skeletal muscle cells. Finally, due to the increasing number of participants in ultra-endurance events, future research should focus on non-elite ultra-cyclers, particularly on preparation and training for ultra-endurance performance. Research is also needed exploring the long-term effects of ultra-cycling and on the persistence of post-acute metabolic modifications..

## Data Availability

The datasets used and/or analyzed during the current study are available from the corresponding author upon a reasonable request.
